# A Homogeneous Anisotropic Hardening Model in Plane Stress State for Sheet Metal under Nonlinear Loading Paths

**DOI:** 10.3390/ma16031151

**Published:** 2023-01-29

**Authors:** Haihui Zhu, Yanli Lin, Kelin Chen, Zhubin He, Shijian Yuan

**Affiliations:** 1 School of Mechanical Engineering, Dalian University of Technology, Dalian 116024, China; 2 Department of Integrated Systems Engineering, The Ohio State University, Columbus, OH 43210, USA; 3 National Key Laboratory for Precision Hot Processing of Metals, Harbin Institute of Technology, Harbin 150001, China

**Keywords:** homogeneous anisotropic hardening model, sheet metal, nonlinear loading path, plane stress

## Abstract

In sheet metal forming, the material is usually subjected to a complex nonlinear loading process, and the anisotropic hardening behavior of the material must be considered in order to accurately predict the deformation of the sheet. In recent years, the homogeneous anisotropic hardening (HAH) model has been applied in the simulation of sheet metal forming. However, the existing HAH model is established in the second-order stress deviator space, which makes the calculation complicated and costly, especially for a plane stress problem such as sheet metal forming. In an attempt to reduce the computational cost, an HAH model in plane stress state is proposed, and called the HAH-2d model in this paper. In the HAH-2d model, both the stress vector and microstructure vector contain only three in-plane components, so the calculation is significantly simplified. The characteristics of the model under typical nonlinear loading paths are analyzed. Additionally, the feasibility of the model is verified by the stress–strain responses of DP780 and EDDQ steel sheets under different two-step uniaxial tension tests. The results show that the HAH-2d model can reasonably reflect the Bauschinger effect and the permanent softening effect in reverse loading, and the latent hardening effect in cross loading, while the predictive accuracy for cross-loading softening remains to be improved. In the future, the HAH-2d model can be further modified to describe more anisotropic hardening behaviors and applied to numerical simulations.

## 1. Introduction

With increasing requirements for lightweight materials in the aerospace, aviation, and automotive industries, integrated thin-walled components with complex shapes are finding broader applications [1,2]. During the forming of an integrated component, the material often undergoes complex nonlinear loading paths, even including multi-step pre-deformation. The deformation behavior of sheet metal often exhibits strong path dependence [3,4,5] and pronounced anisotropic hardening behaviors in nonlinear loading paths; these may be, for example, the Bauschinger effect, the permanent softening effect and the latent hardening effect [6,7].

The Bauschinger effect is the phenomenon wherein the yield stress under reverse loading decreases after a certain amount of pre-deformation [8,9]; the permanent softening effect is another phenomenon during reverse loading wherein the reloading flow stress remains lower than the flow stress of monotonic loading [10]. Meanwhile, there is a wide range of strain path changes between monotonic loading and reverse loading. For some materials, the reloading flow stress at an angle with respect to the first loading may overshoot the monotonic stress–strain curve; this phenomenon is referred to the latent hardening effect [11,12].

Practically, the anisotropic hardening behavior significantly affects the spring-back and forming limit [13,14,15], and the reduction of spring-back and the avoidance of necking or fracture are of paramount importance in the forming of an integrated component. In order to guide process formulation and reduce the cost of process development, it is necessary to predict the deformation process and optimize the forming process by simulation. At present, the isotropic hardening assumption is commonly used in general simulations of metal forming, i.e., the subsequent yield surface expands uniformly based on the initial yield surface. However, an isotropic hardening model cannot reasonably capture the anisotropic hardening behavior under nonlinear loading paths [16,17]. Therefore, establishing advanced anisotropic hardening models to accurately predict the plastic deformation behavior of thin-walled metals under nonlinear loading conditions is an important research direction [18].

A variety of anisotropic hardening models have been proposed [19,20], for example, the kinematic hardening model established by introducing a back stress tensor into the isotropic hardening model. The kinematic hardening model was first proposed by Prager to capture the Bauschinger effect, wherein the yield surface translates with the deformation but its shape remains the same [21]. Subsequently, some kinematic hardening models with a nonlinear relationship between the increments of back stress and plastic strain were proposed to more accurately capture the flow stress under reverse loading or cyclical loading conditions [22,23]. In fact, the typical hardening behavior of metals is often a mixture of isotropic hardening and kinematic hardening, i.e., the yield surface changes both in size and position. Hence, the mixed hardening mode was proposed [24,25].

As an alternative to the kinematic hardening model, Barlat et al. [26] proposed the homogeneous anisotropic hardening (HAH) model, which is a distortional hardening model without a back stress tensor. In the HAH model, a fluctuating component is added into the traditional yield function to change the shape of yield surface, and a microstructure deviator related to the loading history is introduced to reflect the state of the microstructure. In order to further describe the latent hardening, more variables were introduced into the model [27,28]. More importantly, the HAH model can be regarded as a theoretical framework for the anisotropic hardening model, in which any homogeneous yield function and hardening law can be used, and the yield surface can be distorted depending on the specific loading path [17]. Some researchers have successfully implemented the HAH model in numerical simulations, and the prediction accuracy has improved clearly [29,30,31].

More recently, in order to capture complex and varied anisotropic hardening behavior and improve prediction accuracy, more parameters were introduced, for example, the HAH-20 model (using, at most, 23 coefficients [17]) and the HEXAH model (using, at most, 15 coefficients [32]). On the other hand, in order to reduce the computational cost in numerical simulation, some efficient algorithms were implemented, including a multi-step return-mapping algorithm for the HAH model [33], a fully implicit numerical algorithm that can solve a complete set of residuals [34], and a fast and robust stress-update algorithm based on the general cutting-plane method [35].

It should be pointed out that the HAH model and these improvements were proposed based on the second-order stress deviator with six independent components, corresponding to the fully 3D (three dimensional) stress state. However, in the plastic forming of thin-walled components, the normal stress is usually much smaller than the in-plane principal stresses, which means that the stress condition can be simplified to the plane stress state with only three components. Therefore, shell elements and material constitutive models for plane stress states are widely used in numerical simulations of sheet metal forming. When the HAH model is selected to analyze the deformation of sheet metals under nonlinear loading paths, it is required to transform the in-plane stress components to a stress deviator, then calculate the strain increments and new stress deviator after a deformation increment using an iterative method, and lastly, transform the new stress deviation tensor into in-plane stress components for output. Obviously, the plane stress problem is transformed into a more complex 3D stress problem, which makes the calculation complicated and costly. Therefore, it is of great significance to establish an anisotropic hardening model in plane stress state to analyze the deformation of sheet metals subjected to complex loading.

In this paper, an anisotropic hardening model for plane stress problems such as thin-walled metal forming will be proposed based on the framework of the HAH model. For convenience, the proposed model is named the HAH-2d model in this work. In the meantime, the performance of the HAH-2d model in predicting the evolution of yield loci and the stress–strain curve will be analyzed under typical nonlinear loading paths. Moreover, the new model will be verified by experimental results from two-step uniaxial tension tests.

## 2. Fundamentals of the HAH Model

The original HAH model was proposed by Barlat et al. in the second-order stress deviator space [26]. The yield function, as well as the plastic potential, is as follows:

(1) $$\Phi \left(s\right)={\left[\phi^{q}+\phi_{h}^{q}\right]}^{\frac{1}{q}}={\left[\phi^{q}(s)+f_{1}^{q}{\left|\hat{h}:s-\left|\hat{h}:s\right|\right|}^{q}+f_{2}^{q}{\left|\hat{h}:s+\left|\hat{h}:s\right|\right|}^{q}\right]}^{\frac{1}{q}}=\bar{\sigma }\left(\bar{\varepsilon }\right)$$

where, **s** and $\hat{h}$ are the stress deviator and microstructure deviator, respectively, and “:” denotes the double dot product.

The yield function Φ(s) consists of a stable component ϕ and a fluctuating component $\phi_{h}$. Any yield function may be used as the stable component after being reduced to a homogeneous function of degree 1 with the form of $\phi (s)=\bar{\sigma }$. $\bar{\sigma }\left(\bar{\varepsilon }\right)$ is a hardening law, where $\bar{\sigma }$ and $\bar{\varepsilon }$ are the equivalent stress and equivalent plastic strain, respectively. $f_{1}$ and $f_{2}$ are state variables related to deformation history leading to the distortion of the yield surface, and *q* is a constant exponent. If $f_{1}=f_{2}=0$, Equation (1) reduces to a conventional yield function, $\Phi (s)=\phi \left(s\right)=\bar{\sigma }\left(\bar{\varepsilon }\right)$.

The microstructure deviator $\hat{h}$, a normalized tensorial state variable, is proposed to capture the deformation history and reflect the microstructure evolution of the material. It defines an axis, not a direction; namely, $\hat{h}$ and $-\hat{h}$ represent the same microstructural state. Its initial value ${\hat{h}}^{0}$ is defined as the normalized stress deviator ${\hat{s}}^{0}$ corresponding to the initial yield, as in the following equation, where the factor 8/3 is used for convenience [26,27].

(2) $${\hat{h}}^{0}={\hat{s}}^{0}=\frac{{\hat{s}}^{0}}{\sqrt{\frac{8}{3}s_{ij}^{0}s_{ij}^{0}}}$$

If the material is reloaded in a different stress state, $\hat{h}$ will rotate gradually towards the new stress deviator **s** when cosχ≥0, or towards −**s** when cosχ<0. Additionally, cosχ determined by Equation (3) is the cosine of the angle between **s** and $\hat{h}$, representing the variation of the loading path [27,28]. Monotonic, reverse and cross-loading sequences are, respectively, represented by cosχ = 1, −1 and 0.

(3) $$\cos\chi =\frac{s:\hat{h}}{\left\|s\right\|\cdot \left\|\hat{h}\right\|}$$

where, $\left\|s\right\|=\sqrt{s:s}=\sqrt{s_{ij}s_{ij}}$ denotes the norm of the second-order tensor **s**.

## 3. HAH-2d Model in Plane Stress State

### 3.1. Stress Vector and Microstructure Vector

During the forming process of sheet metal, the material is mainly in plane stress state; the three in-plane stress components can be simply expressed as a stress vector $\sigma ={\left(\sigma_{11},\;\sigma_{22},\;\sigma_{12}\right)}^{T}$, while the other stress components such as $\sigma_{33}$, $\sigma_{13}$ and $\sigma_{23}$ are equal to 0. In order to describe the microstructure evolution of the sheet metal under plane stress conditions, a normalized microstructure vector $\hat{H}={\left({\hat{H}}_{11},\;{\hat{H}}_{22},\;{\hat{H}}_{12}\right)}^{T}$ is defined in terms of stress vector in this paper. Its initial value ${\hat{H}}^{0}$ also corresponds to the normalized stress vector ${\hat{\sigma }}^{0}$ leading to the first increment of plastic deformation.

(4) $${\hat{H}}^{0}={\hat{\sigma }}^{0}=\frac{\sigma^{0}}{\sqrt{\sigma^{0}:\sigma^{0}}}$$

Considering that the plastic deformation of metals is driven by the stress deviator **s**, it is still necessary to discuss the variation of loading path based on stress deviator when analyzing plastic deformation processes under plane stress conditions. The second-order deviatoric tensors, **s** and **h**, corresponding to the stress vector σ and the microstructure vector $\hat{H}$, can be obtained through the following two equations, respectively.

(5) $$s=\left(\begin{array}{ccc} \sigma_{11} & \sigma_{12} & 0\\ \sigma_{12} & \sigma_{22} & 0\\ 0 & 0 & 0 \end{array}\right)-\frac{\sigma_{11}+\sigma_{22}}{3}\left(\begin{array}{ccc} 1 & 0 & 0\\ 0 & 1 & 0\\ 0 & 0 & 1 \end{array}\right)$$

(6) $$h=\left(\begin{array}{ccc} {\hat{H}}_{11} & {\hat{H}}_{12} & 0\\ {\hat{H}}_{12} & {\hat{H}}_{22} & 0\\ 0 & 0 & 0 \end{array}\right)-\frac{{\hat{H}}_{11}+{\hat{H}}_{22}}{3}\left(\begin{array}{ccc} 1 & 0 & 0\\ 0 & 1 & 0\\ 0 & 0 & 1 \end{array}\right)$$

Then, the double dot product s:h can be expressed as:

(7) $$s:h=\frac{1}{3}\left(2\sigma_{11}{\hat{H}}_{11}+2\sigma_{22}{\hat{H}}_{22}-\sigma_{11}{\hat{H}}_{22}-\sigma_{22}{\hat{H}}_{11}+6\sigma_{12}{\hat{H}}_{12}\right)=\sigma^{T}D\hat{H}$$

where the matrix $D=\frac{1}{3}\left(\begin{array}{ccc} 2 & -1 & 0\\ -1 & 2 & 0\\ 0 & 0 & 6 \end{array}\right)$.

Therefore, the value of cosχ can be calculated through vectors σ and $\hat{H}$, as follows:

(8) $$\cos\chi =\frac{\sigma^{T}D\hat{H}}{\sqrt{\sigma^{T}D\sigma }\sqrt{{\hat{H}}^{T}D\hat{H}}}$$

Moreover, $\hat{H}$ defines an axis in the vector space of plane stress. When the sheet metal undergoes loading-path changes, $\hat{H}$ will remain the same if cosχ=±1, or rotate gradually towards the new stress vector σ if 0≤cosχ<1, or rotate towards −σ if −1≤cosχ<0. In this work, a possible evolution for $\hat{H}$ is given as follows:

(9) $$\left\{ \begin{array}{l} \;d\hat{H}=k\left(\hat{\sigma }-\hat{H}\right)d\bar{\varepsilon },\;\cos\chi \geq 0\\ \;d\hat{H}=k\left(-\hat{\sigma }-\hat{H}\right)d\bar{\varepsilon },\;\cos\chi <0 \end{array}\right.$$

(10) $$H^{i+1}={\hat{H}}^{i}+d\hat{H}$$

(11) $${\hat{H}}^{i+1}=\frac{H^{i+1}}{\sqrt{{\left(H_{11}^{i+1}\right)}^{2}+{\left(H_{22}^{i+1}\right)}^{2}+{\left(H_{12}^{i+1}\right)}^{2}}}$$

in which $\hat{\sigma }$ is the normalized stress vector of σ, *k* is a constant that controls the rate of rotation, and $d\bar{\varepsilon }$ is the increment of equivalent strain $\bar{\varepsilon }$, which can be calculated according to the plastic work increment $dW^{p}$:

(12) $$\bar{\sigma }d\bar{\varepsilon }=\sigma \cdot d\varepsilon =dW^{p}$$

(13) $$d\varepsilon ={\left(d\varepsilon_{11},\;d\varepsilon_{22},\;d\gamma_{12}\right)}^{T}$$

where dε is the plastic strain increment vector, $d\varepsilon_{11}$ and $d\varepsilon_{22}$ are strain increments along directions 1 and 2, and $d\gamma_{12}$ is the shear strain increment.

**Figure 1:**
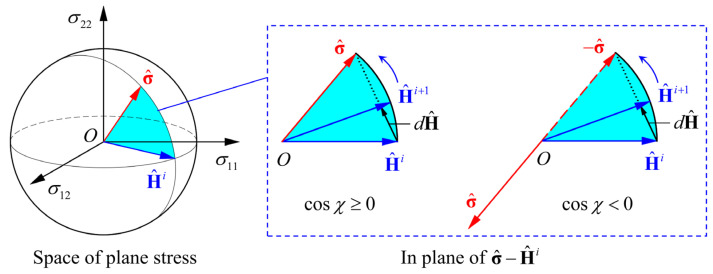
Schematic diagram of the evolution of microstructure vector $\hat{H}$.

The evolution of $\hat{H}$ is shown schematically in Figure 1. $\hat{H}$ rotates in the plane determined by ${\hat{H}}^{i}$ and $\hat{\sigma }$, and finally, $\hat{H}$ takes the direction of $\hat{\sigma }$ or $-\hat{\sigma }$.

### 3.2. Formulation of the HAH-2d Model

#### 3.2.1. Distortional Yield Function

The distortional yield function of the HAH model in the plane stress space (HAH-2d model) is as follows:

(14) $$\Phi \left(\sigma \right)=\frac{1}{F_{L}}{\left[\phi^{q}+\phi_{h}{}^{q}\right]}^{\frac{1}{q}}=\frac{1}{F_{L}}{\left[\phi^{q}(\sigma )+f_{1}^{q}{\left|\sigma_{p}-\left|\sigma_{p}\right|\right|}^{q}+f_{2}^{q}{\left|\sigma_{p}+\left|\sigma_{p}\right|\right|}^{q}\right]}^{\frac{1}{q}}=\bar{\sigma }\left(\bar{\varepsilon }\right)$$

(15) $$\sigma_{p}=\left\|s\right\|\cos\chi =\frac{\sigma^{T}D\hat{H}}{\sqrt{{\hat{H}}^{T}D\hat{H}}}$$

The stable component ϕ can be any homogeneous yield function of degree 1 for plane stress. The fluctuating component $\phi_{h}$ is adopted to cover the Bauschinger and permanent softening effects, and the multiplier component $1/F_{L}$ is used to capture the latent hardening effect. Correspondingly, Φ(σ) is also a homogeneous function of degree 1, i.e., Φ(aσ)=aΦ(σ) holds for any real number *a*. In addition, $\sigma_{p}$ is the projection of the stress deviator **s** in the direction of the microstructure deviator **h**.

It is important to note that the associated flow rule is applied in this paper, the yield function also serves as the plastic potential. In other words, the plastic strain increment vector dε is defined as

(16) $$d\varepsilon =\frac{\partial \Phi (\sigma )}{\partial \sigma }d\lambda$$

where dλ≥0 is the plastic multiplier.

#### 3.2.2. Bauschinger Effect and Permanent Softening Effect

**Figure 2:**
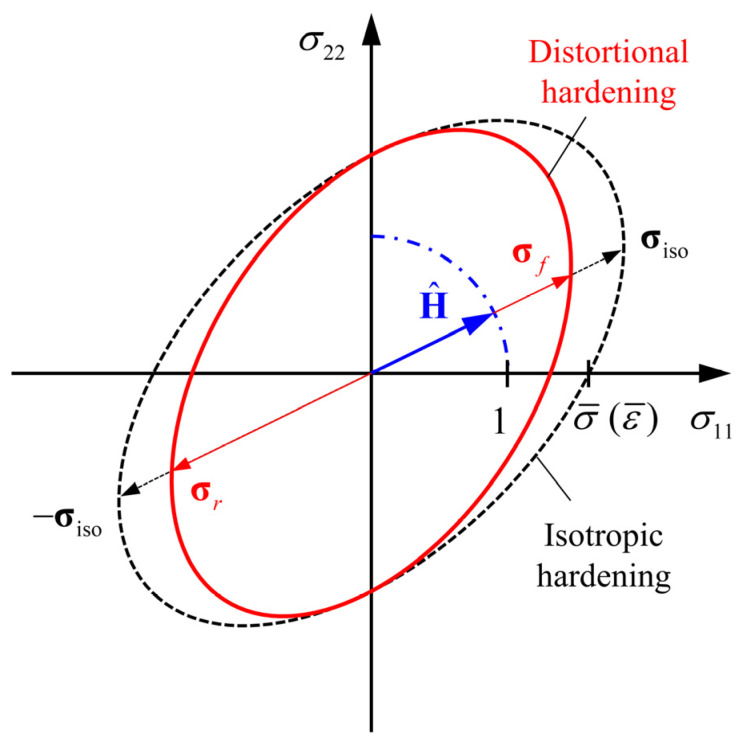
Schematic diagram of yield loci in $\sigma_{11}-\sigma_{22}$ plane with $f_{1}>0$, $f_{2}>0$ and $F_{L}=1$.

A schematic diagram of yield loci in the $\sigma_{11}-\sigma_{22}$ plane with $f_{1}>0$, $f_{2}>0$ and $F_{L}=1$ is shown in Figure 2. $\sigma_{f}$ and $\sigma_{r}$ are yield stress vectors on the current distortional yield locus along the positive and negative directions of $\hat{H}$, respectively, and $\sigma_{\mathrm{iso}}$ is the yield stress vector on the isotropic yield locus. State variables $g_{1}$ and $g_{2}$ are defined as Equation (17) to represent the relationship between $\sigma_{f}$, $\sigma_{r}$ and $\sigma_{\mathrm{iso}}$, and the parameters $f_{1}$ and $f_{2}$ in Equation (14) can be expressed as Equation (18).

(17) $$\left\{ \begin{array}{l} g_{1}=-\sigma_{r}/\sigma_{\mathrm{iso}}\leq 1\\ g_{2}=\sigma_{f}/\sigma_{\mathrm{iso}}\leq 1 \end{array}\right.$$

(18) $$f_{i}=\frac{\sqrt{6}}{4}{\left(g_{i}{}^{-q}-1\right)}^{\frac{1}{q}},\;i=1,\;2$$

For a sheet metal exhibiting the Bauschinger effect, the normalized yield surface in the opposite direction of loading tends to contract during forward loading, and the value of $f_{1}$ should increase from 0 towards a finite value. As a result, the yield stress of reverse loading is reduced compared with the final forward-loading stress, which is the Bauschinger effect. During reverse loading, the flat yield surface at the side of reverse loading tends to recover the yield surface determined by the isotropic hardening function $\phi (\sigma )=\bar{\sigma }$. At the same time, the yield surface opposite to the reverse loading contracts, and $f_{2}$ starts to increase in the same way as $f_{1}$. If the yield surface at the side of reverse loading cannot recover to the level of isotropic hardening, the permanent softening effect will occur during reverse loading correspondingly.

A possible evolution for $g_{1}$ and $g_{2}$ is given in Equation (19) which has been successfully applied in existing HAH models [26,27,28]. State variables $g_{3}$ and $g_{4}$ represent the maximum saturation values of $g_{2}$ and $g_{1}$, respectively. Material coefficients *k*_1_ and *k*_2_ control the evolution rate of $g_{1}$ and $g_{2}$, and *k*_3_ controls their lower bound; $k_{4}$ controls the minimum value of $g_{3}$ and $g_{4}$, and $k_{5}$ controls their evaluation rate. Meanwhile, the value of $k_{4}$ is usually slightly less than 1, according to the permanent softening effect. When the initial values of $g_{1}$ and $g_{2}$ are both 1 and $k_{1}=k_{2}=0$, the ability to describe the Bauschinger effect will be suppressed, and when $g_{3}=g_{4}=1$ and $k_{4}=1$ (or $k_{5}=0$), the ability to describe the permanent softening effect will be suppressed.

(19) $$\begin{array}{l} \mathrm{If}\;\cos\chi \geq 0\\ \frac{dg_{1}}{d\bar{\varepsilon }}=k_{2}\left(k_{3}\frac{\bar{\sigma }\left(0\right)}{\bar{\sigma }\left(\bar{\varepsilon }\right)}-g_{1}\right)\\ \frac{dg_{2}}{d\bar{\varepsilon }}=k_{1}\frac{g_{3}-g_{2}}{g_{2}}\\ \frac{dg_{4}}{d\bar{\varepsilon }}=k_{5}\left(k_{4}-g_{4}\right) \end{array}\begin{array}{l} \mathrm{If}\;\cos\chi <0\\ \frac{dg_{1}}{d\bar{\varepsilon }}=k_{1}\frac{g_{4}-g_{1}}{g_{1}}\\ \frac{dg_{2}}{d\bar{\varepsilon }}=k_{2}\left(k_{3}\frac{\bar{\sigma }\left(0\right)}{\bar{\sigma }\left(\bar{\varepsilon }\right)}-g_{2}\right)\\ \frac{dg_{3}}{d\bar{\varepsilon }}=k_{5}\left(k_{4}-g_{3}\right) \end{array}$$

#### 3.2.3. Latent Hardening Effect

For some materials, the flow stress during reloading may overshoot the stress during monotonic loading under the condition of the same equivalent strain. The phenomenon of overshooting was explained as the latent hardening effect. Additionally, the degree of overshooting is usually maximum for cross loading, in which the angle between the two corresponding normalized stress deviators ${\hat{s}}_{1}$ and ${\hat{s}}_{2}$ is 90° (${\hat{s}}_{1}:{\hat{s}}_{2}=0$). As the degree of plastic deformation increases in the second step, $\hat{h}$ rotates gradually towards ${\hat{s}}_{2}$ or $-{\hat{s}}_{2}$.

Suppose $s_{v}$ and ${s^{\prime }}_{v}$ are the stress deviators vertical to $\hat{h}$ on the distortional yield surface and the isotropic one, respectively. Additionally, the corresponding plane stress vectors are $\sigma_{v}$ and ${\sigma^{\prime }}_{v}$. Then, a state variable $g_{L}$ can be defined as Equation (20), which represents that the distortional yield surface at cosχ=0 expands to $g_{L}$ times the yield surface under monotonic loading, and its minimum value is 1.

(20) $$g_{L}=\frac{\phi \left(\sigma_{v}\right)}{\phi \left({\sigma^{\prime }}_{v}\right)}=\frac{s_{v}}{{s^{\prime }}_{v}}$$

**Figure 3:**
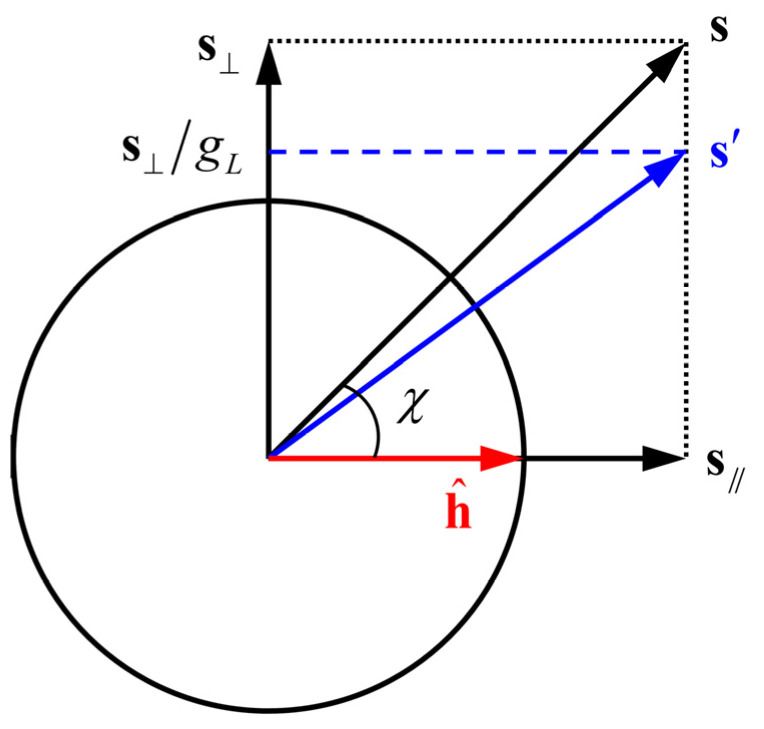
Schematic of the decomposition and composition of stress deviator [28]. Redraw with reference to International Journal of Plasticity, Vol 58, Barlat, F.; Vincze, G.; Grácio, J.J.; Lee, M.G.; Rauch, E.F.; Tomé, C.N., Enhancements of homogenous anisotropic hardening model and application to mild and dual-phase steels, 201–218, Copyright Elsevier (2014), with permission from Elsevier.

Because the degree of the latent hardening effect is different under different strain path changes, it is important to determine the yield surface expansion for arbitrary χ. The stress deviator **s** can be decomposed into $s_{∥}$ parallel to $\hat{h}$ and $s_{⊥}$ perpendicular to $\hat{h}$, as shown in Figure 3 [28]. Considering that the latent hardening effect is mainly caused by the vertical component $s_{⊥}$, a new deviator $s^{\prime }$ is composed as

(21) $$s^{\prime }=s_{⊥}/g_{L}+s_{∥}$$

Then, the expansion ratio of the yield surface for arbitrary χ can be represented by

(22) $$F_{L}=\frac{\left\|s\right\|}{\left\|s^{\prime }\right\|}=\frac{g_{L}}{\sqrt{\left(g_{L}{}^{2}-1\right)\cos^{2}\chi +1}}$$

Thus, the surface of $\phi \left(\sigma \right)/F_{L}=\bar{\sigma }\left(\bar{\varepsilon }\right)$ expands faster than that of $\phi \left(\sigma \right)=\bar{\sigma }\left(\bar{\varepsilon }\right)$ under any strain path change except cosχ=±1. Furthermore, the evolution equation of $g_{L}$ is as follows [27]:

(23) $$\frac{dg_{L}}{d\bar{\varepsilon }}=k_{L}\left[\left(\frac{\bar{\sigma }\left(\bar{\varepsilon }\right)-\bar{\sigma }\left(0\right)}{\bar{\sigma }\left(\bar{\varepsilon }\right)}\right)\left(\sqrt{L(1-\cos^{2}\chi )+\cos^{2}\chi }-1\right)+1-g_{L}\right]$$

where *L* and $k_{L}$ are constants controlling the upper bound and evolution rate of $g_{L}$, respectively. When the initial value of $g_{L}$ is 1 and *L* = 1, $F_{L}$ will be identical to 1, and the latent hardening effect is suppressed.

### 3.3. Coefficient Identification

It is recommended to identify the coefficients in the HAH-2d model in the following order: yield function ϕ(σ), isotropic hardening law $\bar{\sigma }\left(\bar{\varepsilon }\right)$, and distortional hardening [27]. The coefficients of ϕ(σ) and $\bar{\sigma }\left(\bar{\varepsilon }\right)$ can be determined independently in a conventional way; for example, the coefficients of ϕ(σ) can be calculated based on the yield stresses and anisotropy coefficients along different directions of the sheet, and the coefficients of $\bar{\sigma }\left(\bar{\varepsilon }\right)$ can be obtained by fitting a monotonic stress–strain curve. Additionally, the nine coefficients related to the distortion, namely, *q*, *k*, *k*_1_, *k*_2_, *k*_3_, *k*_4_, *k*_5_, *k_L_* and *L*, can be identified using an optimization method in which the input experimental data should come from reverse-loading and cross-loading tests.

When the stress–strain data of forward-reverse loading are available, coefficients *k*_1_, *k*_2_, *k*_3_, *k*_4_ and *k*_5_ can be determined independently. Otherwise, the five coefficients have to be evaluated together with *k*, *k_L_*, *L* and *q*. In particular, the Bauschinger, permanent softening and latent hardening effects can be expressed simultaneously in the stress–strain curve from a two-step tensile test with −1<cosχ<0, for example, re-tension in the direction orthogonal to the fist tensile direction (cosχ=−0.5). Given that the orthogonal two-step tensile test is easy to carry out, all the coefficients associated with the distortion could be identified based on the stress–strain curve.

## 4. Hardening Behavior Predicted under Typical Nonlinear Loading Paths

In this section, the stress–strain curves and yield locus evolutions of a generic sheet material under several typical nonlinear loading paths were predicted by the HAH-2d model. The typical nonlinear loading paths in the range of −1≤cosχ<1 include reverse loading (cosχ=−1), cross loading (cosχ=0) and two-step tensions conducted at 45° (cosχ=0.25) and 90° (cosχ=−0.5) from the first loading direction.

The initial yield condition of the generic sheet material is assumed to satisfy the Mises isotropic yield criterion, see Equation (24), and the hardening satisfies the Swift hardening law [36], see Equation (25). The material has a Bauschinger effect, permanent softening effect and latent hardening effect simultaneously. The coefficients of the swift hardening law and coefficients associated with the distortion are listed in Table 1.

(24) $$\phi_{\mathrm{Mises}}\left(\sigma \right)=\sqrt{\sigma_{11}^{2}+\sigma_{22}^{2}-\sigma_{11}^{}\sigma_{22}^{}+3\sigma_{12}^{2}}=\bar{\sigma }$$

(25) $$\bar{\sigma }=K{\left(\bar{\varepsilon }+\varepsilon_{0}\right)}^{n}$$

where *K*, $\varepsilon_{0}$ and *n* are material coefficients.

### 4.1. Reverse Loading

**Figure 4:**
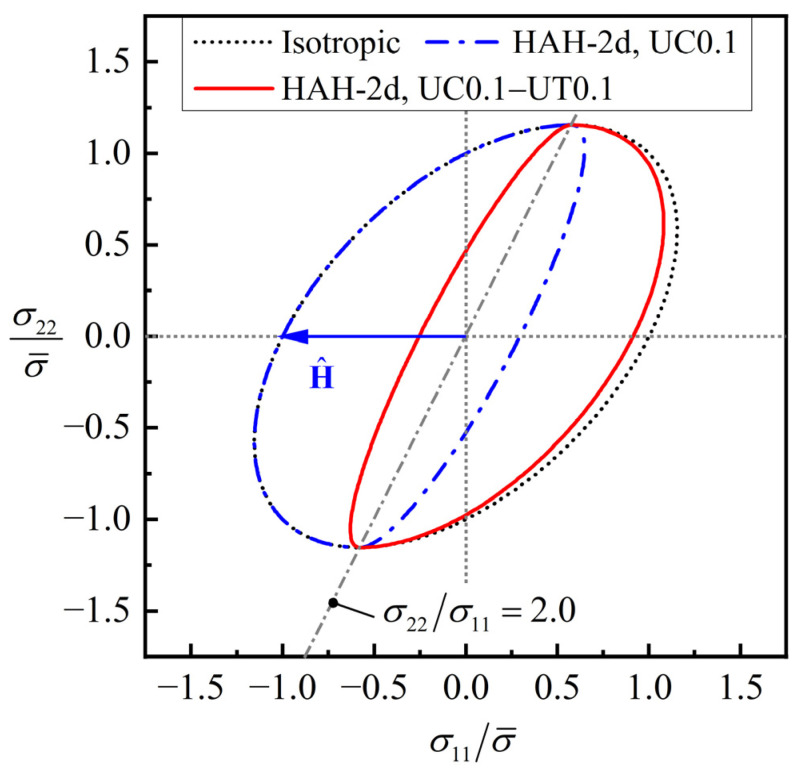
Evolution of the normalized yield locus of the generic material predicted by the HAH-2d model in a uniaxial compression-tension loading path.

The evolution of the normalized yield locus of the generic material, predicted by the HAH-2d model under a reverse loading, namely, uniaxial compression (UC) to true strain of 0.10, followed by uniaxial tension (UT) in the reverse direction, is shown in Figure 4. The microstructure vector $\hat{H}$ maintains (−1, 0, 0) during the whole deformation. The normalized yield loci in $\sigma_{11}-\sigma_{22}$ plane are divided into left and right sides by the line $\sigma_{22}/\sigma_{11}=2.0$. This line is chosen because the two stress deviators corresponding to stress vectors ${\left(1,\;2,\;0\right)}^{T}$ and ${\left(1,\;0,\;0\right)}^{T}$ are orthogonal. During the compression step, the yield locus on the left side remains unchanged, while the yield locus on the right side contracts inward, which will result in a reduction in re-loading yield stress when tension occurs in the opposite direction. As the tensile strain increases in the second step, the yield locus on the left side contracts inward instead. The yield locus on the right side recovers towards the isotropic hardening yield, but not completely, because of the permanent softening effect.

**Figure 5:**
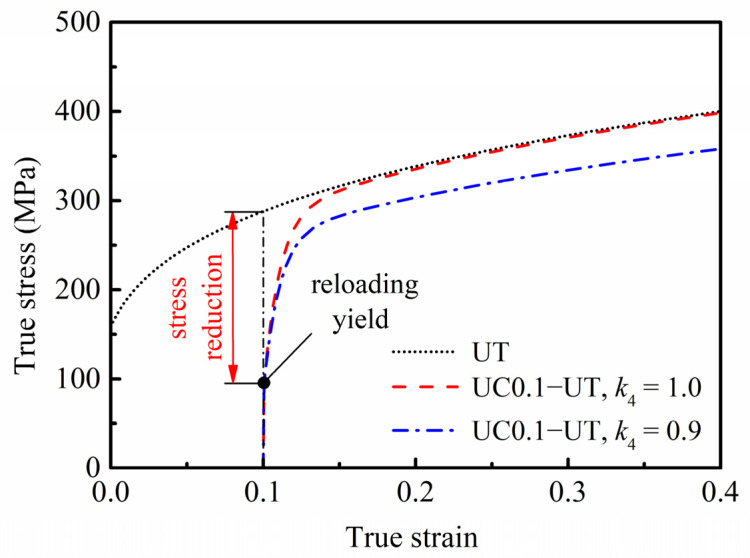
True stress–strain curves of the generic material predicted by the HAH-2d model in a uniaxial compression-tension loading path.

The predicted stress–strain curve during reverse loading is given in Figure 5. The reloading yield stress after compression pre-deformation is significantly lower than the monotonic flow stress under the same deformation level, which is the Bauschinger effect. When *k*_4_ = 0.9, the stress will not reach the monotonic uniaxial tensile curve, which is the permanent softening effect. Additionally, the permanent softening phenomenon disappears when *k*_4_ = 1.0. Therefore, the HAH-2d model can describe the Bauschinger effect and permanent softening effect during reverse loading.

**Figure 6:**
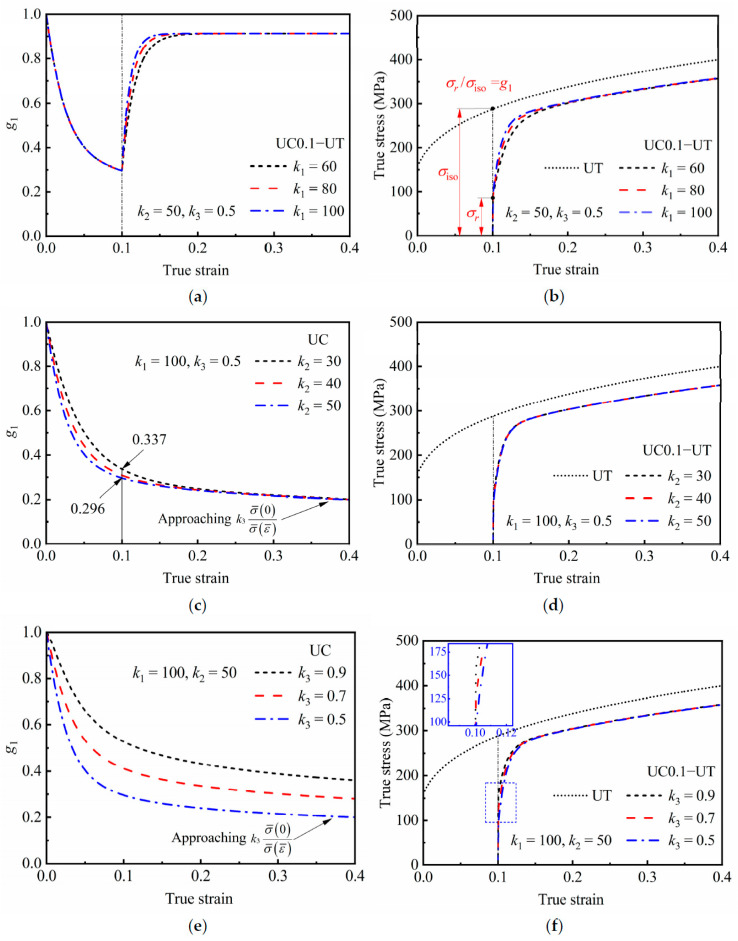
Predicted responses of the Bauschinger effect during reverse loading; evaluation of *g*_1_ during (**a**) UC0.1−UT loading under different *k*_1_; (**c**) UC loading under different *k*_2_; and (**e**) UC loading under different *k*_3_; and stress–strain curves during UC0.1−UT loading under (**b**) different *k*_1_; (**d**) different *k*_2_ and (**f**) different *k*_3_.

The sensitivity of the HAH-2d model to parameters *k*_1_, *k*_2_, and *k*_3_ should be considered when predicting the Bauschinger effect during reverse loading. The state of the yield surface associated with the Bauschinger effect is reflected by state variables *g*_1_ and *g*_2_, and their evolutions follow the same rule. Therefore, the effects of *k*_1_, *k*_2_, and *k*_3_ on the predicted responses, such as the evaluations of *g*_1_ and the stress–strain curves, were analyzed as shown in Figure 6. It can be found from Figure 6a,b that *k*_1_ affects the evaluation rate of *g*_1_ during reloading. The greater *k*_1_ is, the greater the increase rate of *g*_1_, and the faster the flow stress reaches the saturation state. Figure 6c shows that *k*_2_ affects the evaluation rate of *g*_1_ during preloading. However, the corresponding stress–strain curves of reloading under the condition of 30 ≤ *k*_2_ ≤ 50, *k*_1_ = 100 and *k*_3_ = 0.5 are almost coincident, as shown in Figure 6d. The reason is that there is little difference in *g*_1_ after a pre-strain of 0.1, and the difference is quickly covered by the evolution during reverse loading. For the same reason, the difference among the curves in Figure 6f under different *k*_3_ is not obvious too. Even so, it should be noted that the value of *g*_1_ during preloading and the reloading yield stress are significantly affected by *k*_3_; the smaller the value of *k*_3_, the smaller the value of *g*_1_ and the reloading yield stress.

**Figure 7:**
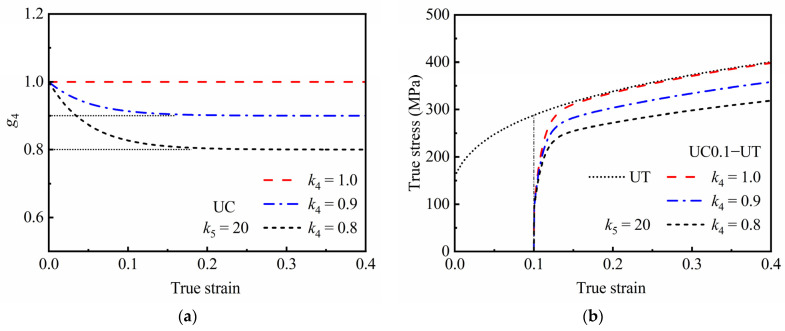

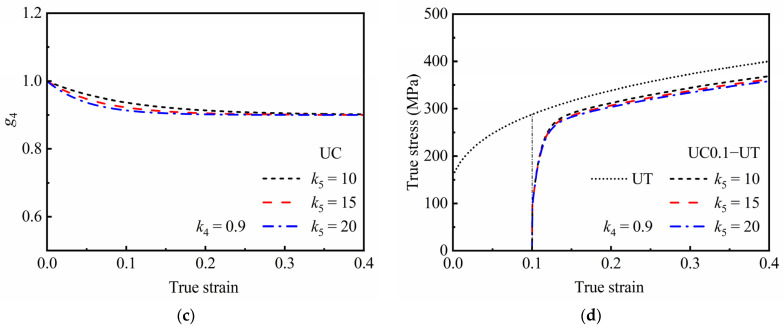
Predicted responses of permanent softening effect during reverse loading; evaluation of *g*_4_ during UC loading under (**a**) different *k*_4_ and (**c**) different *k*_5_; and stress–strain curves during UC0.1−UT loading under (**b**) different *k*_4_ and (**d**) different *k*_5_.

Figure 7 shows the effects of *k*_4_ and *k*_5_ on the predicted responses of the permanent softening effect during reverse loading, where the state variable *g*_4_ represents the maximum saturation value of the reloading stress. It can be seen that the permanent softening effect is mainly affected by *k*_4_, and the smaller the value of *k*_4_, the lower the value of *g*_4_ and the saturation value of reloading stress. In the meantime, when using a value of *k*_4_ less than 1.0, the larger the value of *k*_5_, the faster the value of *g*_4_ approaches the saturation value, and the lower the reloading stress–strain curve will be after a certain pre-strain. Of course, if the pre-strain is large enough, the influence of *k*_5_ on the reloading stress–strain curve will be significantly reduced or may even disappear.

### 4.2. Cross Loading

It is known that the two stress deviators corresponding to ${\hat{\sigma }}_{\mathrm{PT}}={\left(1/\sqrt{5},\;2/\sqrt{5},\;0\right)}^{T}$ and ${\hat{\sigma }}_{\mathrm{UT}}={\left(1,\;0,\;0\right)}^{T}$ are orthogonal to each other, and the corresponding deformation types are plane strain tension and uniaxial tension, respectively. Therefore, the loading sequence of a plane strain tension (PT) followed by a uniaxial tension at an angle of 90° from the first tension direction is a typical cross loading path, and cosχ=0 at the start of the second loading.

**Figure 8:**
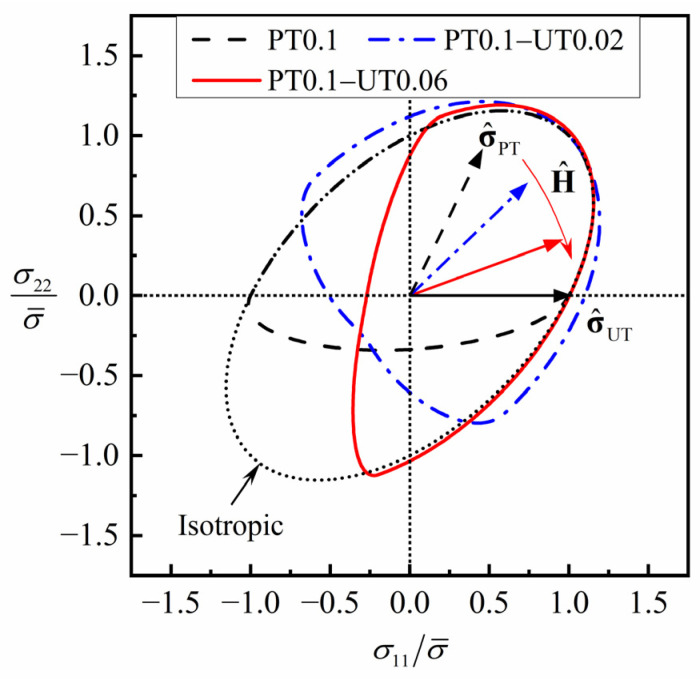
Evolution of the normalized yield locus of the generic material predicted by HAH-2d model in plane strain tension–uniaxial tension.

The evolution of the normalized yield locus of the generic material, predicted by the HAH-2d model during uniaxial tension after plane strain tension to equivalent strain of 0.10, is shown in Figure 8. In the plane strain tension process, there is $\hat{H}={\hat{\sigma }}_{\mathrm{PT}}$, the yield locus above the line $\sigma_{22}=0$ remains unchanged, and the yield locus below the line $\sigma_{22}=0$ contracts inward because of the Bauschinger effect. In the second loading step, $\hat{H}$ rotates gradually towards ${\hat{\sigma }}_{\mathrm{UT}}$ with the increase in tensile strain, and the yield locus near ${\hat{\sigma }}_{\mathrm{UT}}$ expands outward first and then returns to the isotropic hardening yield locus.

**Figure 9:**
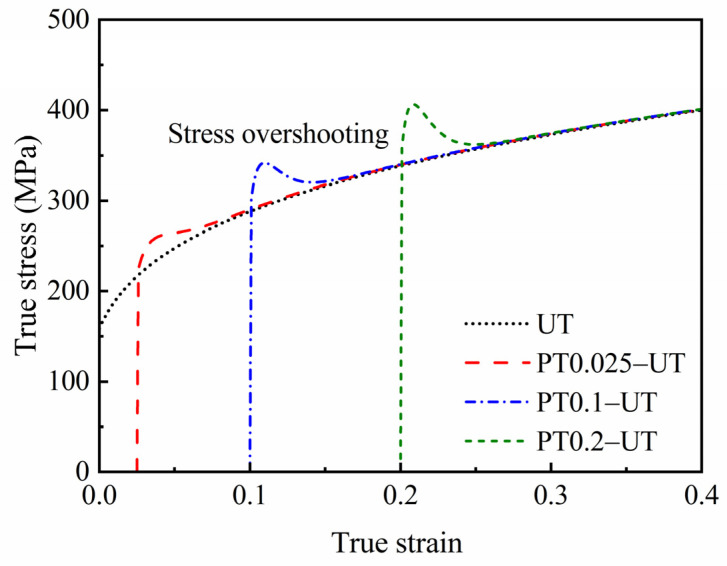
Stress–strain curves predicted by the HAH-2d model in plane strain tension–uniaxial tension with different pre-strain levels.

The uniaxial tensile stress–strain curves in the second loading step after different degrees of plane strain pre-tension are shown in Figure 9. Obvious stress overshooting is observed, and the higher the pre-deformation the greater the overshooting. When the tensile strain reaches about 0.06 in the second step, the tensile stress returns to the level of single uniaxial tension. It indicates that the HAH-2d model can capture the latent hardening in cross loading.

**Figure 10:**
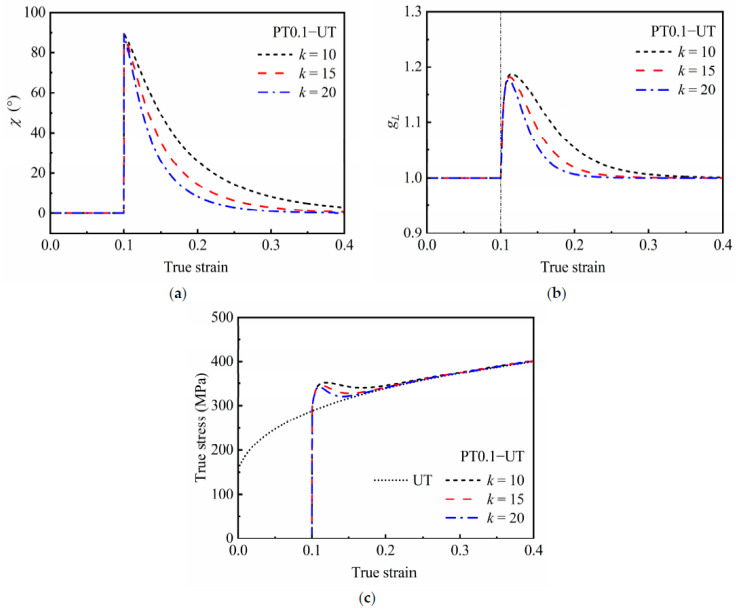
Predicted responses of latent hardening effect during cross loading of PT0.1−UT under different *k*: (**a**) evaluation of *χ*, (**b**) evaluation of *g_L_* and (**c**) stress–strain curves.

The latent hardening effect predicted by the HAH-2d model during cross loading is affected mainly by parameters *k*, *L*, and *k_L_*. Therefore, the sensitivity of the model to these three parameters was analyzed. Figure 10 shows the predicted responses of latent hardening effect under different values of *k* during a typical cross loading, PT0.1−UT. With the increase in the reloading strain, the value of *χ* gradually decreases from 90° to 0°, and the value of *g_L_* rapidly increases to a certain value and then gradually decreases to 1.0. As a result, the phenomenon of stress overshooting is observed. Meanwhile, the larger the value of *k*, the faster the value of *χ* and *g*_4_ decrease, and the faster the overshoot stress recovers.

**Figure 11:**
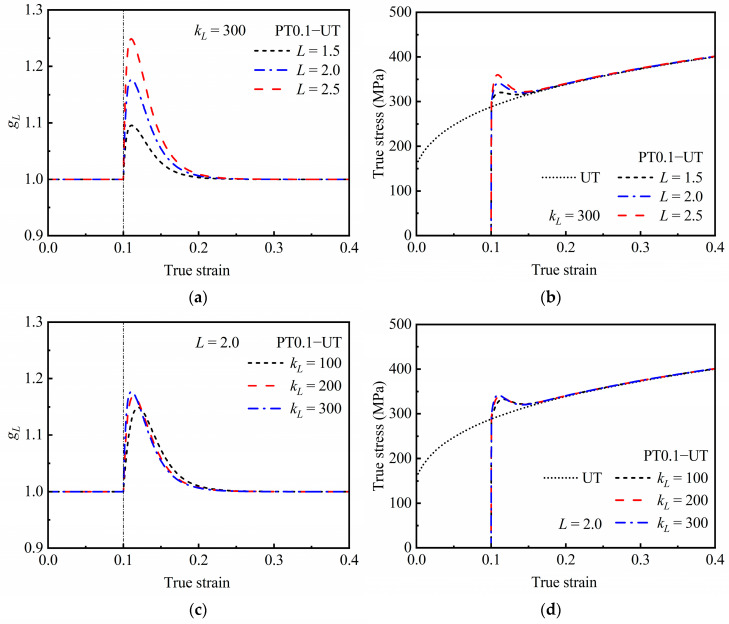
Predicted responses of latent hardening effect during cross loading of PT0.1−UT; evaluation of *g_L_* under (**a**) different *L* and (**c**) different *k_L_*; and stress–strain curves under (**b**) different *L* and (**d**) different *k_L_*.

Figure 11 shows the effects of *L* and *k_L_* on the predicted responses of latent hardening effect during cross loading. It can be seen from Figure 11a,b that the larger the value *L*, the larger *g_L_* can be obtained and the more significant the stress overshooting. On the other hand, the latent hardening effect is also influenced by the value of *k_L_*, as shown in Figure 11c,d. Additionally, the larger the value of *k_L_*, the faster the stress value reaches its peak, and the larger the maximum stress value is. However, the sensitivity of the model to *k_L_* is much lower than that to *L*. In addition, neither *L* nor *k_L_* affects the strain span of the phenomenon of stress overshooting.

### 4.3. Two-Step Uniaxial Tension

**Figure 12:**
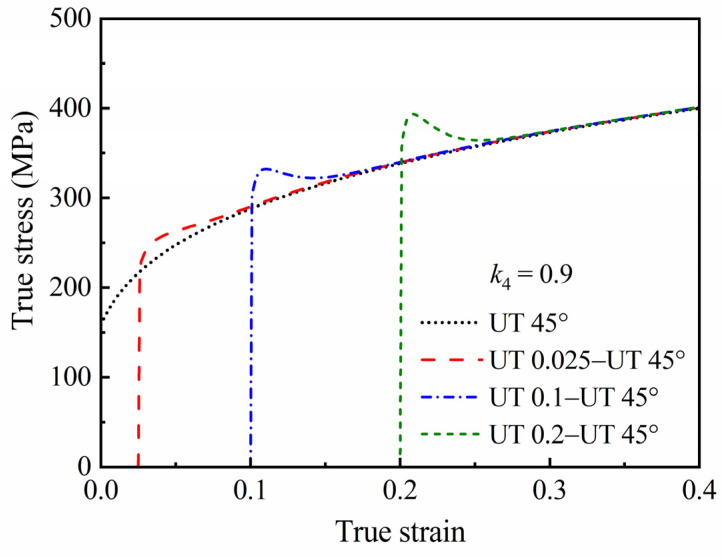
Stress–strain curves predicted by the HAH-2d model in two-step uniaxial tensions with tensile axes at 45° from each other.

**Figure 13:**
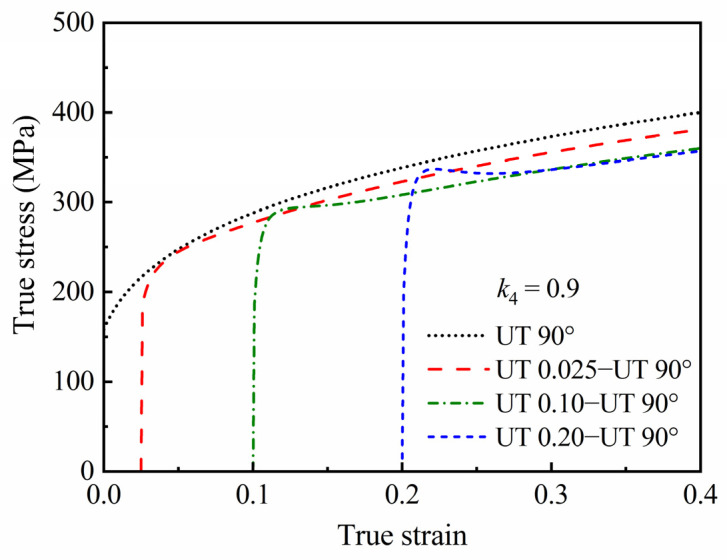
Stress–strain curves predicted by the HAH-2d model in two-step uniaxial tensions with tensile axes at 90° from each other.

Stress–strain curves in two-step uniaxial tensions with tensile axes at 45° and 90° from each other were predicted by the HAH-2d model and are shown in Figure 12 and Figure 13, respectively. The curves with 45° express only latent hardening, while the curves with 90° express the Bauschinger effect, permanent softening and latent hardening at the same time. Therefore, it is better to optimize the coefficients of the HAH-2d model by using the stress–strain data of a two-step uniaxial tension with 90°, or the orthogonal two-step tension mentioned in Section 3.3.

## 5. Model Validation

### 5.1. Determination of Material Coefficients

**Figure 14:**
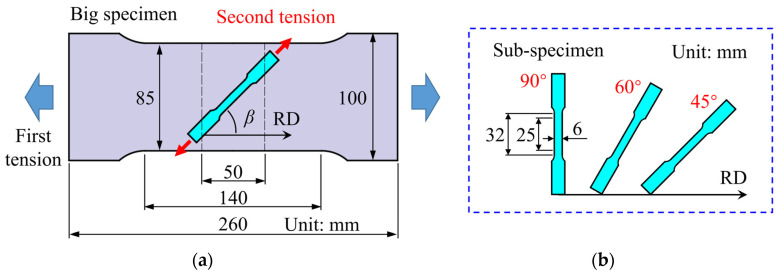
Schematic illustration of the specimens used in the two-step uniaxial tension tests: (**a**) the sub-specimen at the center of the big-specimen and (**b**) directions of the second tension [6].

In this paper, the HAH-2d model will be applied to describe the hardening behavior of DP780 and EDDQ steel sheets with 1.2 mm thickness that were tested by Ha et al. using two-step uniaxial tension tests [6]. As shown in Figure 14, the sub-specimens were cut along different directions at the center of the pre-deformed big-specimen [6]. Additionally, the experimental results from tests consisted first of tension in the rolling direction (RD) and second of tension at 90° (TD, the transverse direction), 60° and 45° from RD were used in this work. Coefficients of the Yld2000-2d anisotropic yield function (see Appendix A) and the Swift hardening law (see Equation (25)) for the two materials are listed in Table 2, where *m* = 6 was adopted as recommended for BCC metals [6].

To quantitatively evaluate the error between the predicted and experimental data, the root mean square error (RMSE) and the average absolute relative error (AARE) were used in this paper. The definition of RMSE and AARE can be expressed by Equations (26) and (27).

(26) $$\mathrm{RMSE}=\sqrt{\frac{1}{N}\sum_{1}^{N}{\left(\sigma_{P}-\sigma_{E}\right)}^{2}}$$

(27) $$\mathrm{AARE}\;(\%)=\frac{1}{N}\sum_{1}^{N}\left|\frac{\sigma_{P}-\sigma_{E}}{\sigma_{E}}\right|\times 100\%$$

where $\sigma_{P}$ is the predicted stress, $\sigma_{E}$ is the experimental stress, and *N* is the number of data.

**Figure 15:**
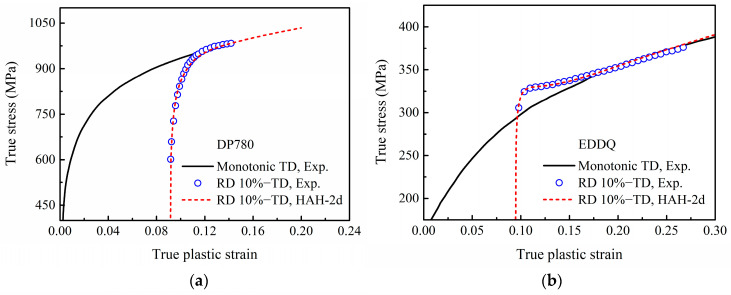
Experimental and predicted stress–strain curves of re-tension in TD with 10% uniaxial pre-strain in RD for: (**a**) DP780 steel and (**b**) EDDQ steel. The experimental data are derived from reference [27]: International Journal of Plasticity, Vol 46, Barlat, F.; Ha, J.; Grácio, J.J.; Lee, M.; Rauch, E.F.; Vincze, G., Extension of homogeneous anisotropic hardening model to cross-loading with latent effects, 130–142, Copyright Elsevier (2013), with permission from Elsevier.

The coefficients associated with the distortion of the HAH-2d model for each material were identified with an optimization method using the stress–strain data from an orthogonal two-step tensile test with 10% pre-strain in RD, as shown in Figure 15. In the optimization method, the objective is to minimize the RMSE. Considering that the DP780 steel expresses no permanent softening and latent hardening, *k*_4_ = 1.0, *k*_5_ = 0, *k_L_* = 0 and *L* = 1.0 were set directly. Similarly, as the EDDQ steel expresses no permanent softening, *k*_4_ = 1.0 and *k*_5_ = 0 were set. In the meantime, the value of *q* was set to 2.0 for convenience. The obtained coefficients of the HAH-2d models for DP780 and EDDQ are listed in Table 3. Additionally, the predicted stress–strain curves are plotted in Figure 15a,b, both of which agree well with the experimental data. Additionally, the values of RMSE for DP780 and EDDQ are 9.81 and 1.85, respectively. Incidentally, the reason for using the experimental data from reference [27] in Figure 15 is that the experimental data used in reference [27] are also from reference [6], but more points are given for this testing condition.

### 5.2. Comparison of Prediction and Experimental Results

In order to validate the HAH-2d model, the hardening curves in two-step uniaxial tensions of DP780 and EDDQ sheets, which consisted of 4% or 10% pre-strain in RD and re-tension at 90° (TD), 60° or 45° from RD, were predicted and compared with the experimental results. Incidentally, the two-step tension with tensile axes at 60° from each other can be called “pseudo cross loading”, because it is close to the ideal cross-loading condition, about 55° between the first and second tensile directions.

**Figure 16:**
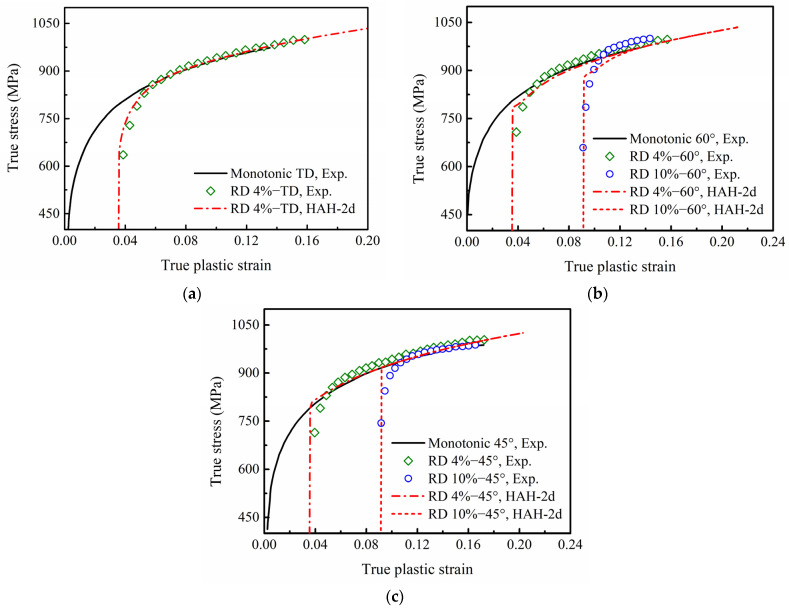
Experimental and predicted stress–strain curves of re-tension at (**a**) 90°, (**b**) 60° and (**c**) 45° from RD with 4% or 10% uniaxial pre-strain in RD for DP780. The experimental data are derived from reference [6]: Mechanics of Materials, Vol 64, Ha, J.; Lee, M.; Barlat, F., Strain hardening response and modeling of EDDQ and DP780 steel sheet under non-linear strain path, 11–26, Copyright Elsevier (2013), with permission from Elsevier.

The predicted stress–strain curves of the HAH-2d model for DP780 under different two-step uniaxial tensions are compared with the experimental curves in Figure 16, and the corresponding prediction errors are listed in Table 4. It can be seen from Figure 16a that the HAH-2d model captures the transient hardening behavior of DP780 fairly well for re-tension in TD after a 4% pre-strain in RD, and the AARE value is only 1.28%. However, the prediction at 60° shown in Figure 16b slightly overestimates the re-loading yield stress and leads to a lower hardening rate. As a result, the values of RMSE and AARE increase to 37.85 and 3.44% under the condition of 10% pre-strain. On the other hand, as shown in Figure 16c, the phenomenon of cross-loading softening was observed in re-loading stress–strain curves at 45°, but the predicted re-loading yield stresses at 45° reach the monotonic curve immediately. Additionally, the high RMSE values of 24.49 and 47.13 are mainly due to the significant difference between the predicted stress and the experimental stress at the beginning of the second tension.

**Figure 17:**
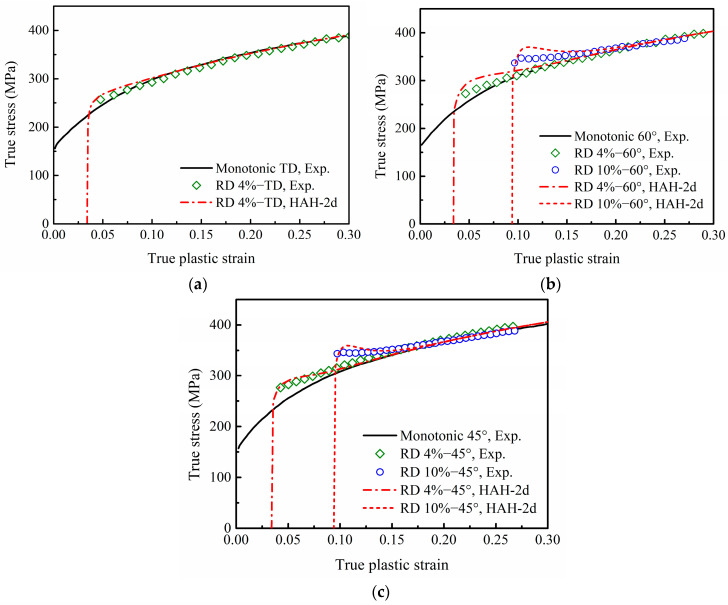
Experimental and predicted stress–strain curves of re-tension at (**a**) 90°, (**b**) 60° and (**c**) 45° from RD with 4% or 10% uniaxial pre-strain in RD for EDDQ. The experimental data are derived from reference [6]: Mechanics of Materials, Vol 64, Ha, J.; Lee, M.; Barlat, F., Strain hardening response and modeling of EDDQ and DP780 steel sheet under non-linear strain path, 11–26, Copyright Elsevier (2013), with permission from Elsevier.

Figure 17a–c show the predicted reloading flow stress curves for EDDQ in different two-step uniaxial tensions with pre-tension in RD and re-tension at 90° (TD), 60° and 45° from RD. The predicted curves capture all the experimental results well, even though there is a little overestimation of the flow stress in the re-tension at 60° from RD. The corresponding prediction errors are listed in Table 5. The maximum values of RMSE and AARE, 9.86 and 2.00%, both occur in the condition of re-tension at 60° after a 10% pre-tension. This indicates that the HAH-2d model can capture anisotropic hardening responses such as flow stress overshooting and strain-hardening stagnation.

## 6. Conclusions

In this work, an HAH model in plane stress state was proposed, and the evolution of the yield loci and the stress–strain curve of a generic material under some typical nonlinear loading paths was analyzed using this model. Furthermore, the model was validated by experimental results from two-step uniaxial tension tests. The conclusions are as follows:

(1) The HAH-2d model was proposed in plane stress state. Both the loading stress vector and microstructure vector contain only three in-plane components. Compared with the HAH model in fully 3D space, the transformation between the plane stress vector and the second-order stress deviator and the complicated calculation in the stress deviator space can be avoided in the HAH-2d model. The computational cost in finite element applications is therefore reduced.

(2) The Bauschinger effect and permanent softening effect in reverse loading and the latent hardening effect in cross loading were investigated in this paper. The evolution of the yield surface and the stress–strain curve of the sheet metal with these effects can be predicted by the HAH-2d model.

(3) The HAH-2d model can reasonably predict experimental phenomena such as the reduction of re-loading yield stress in two-step loading with cosχ<0 and stress overshooting in cross loading. However, the phenomenon of cross-loading softening observed in two-step loading with 0≤cosχ<1 cannot be captured by the model.

## Figures and Tables

**Table 1:** Coefficients of the generic material.

Coefficients	Swift Hardening Law				Coefficients Associated with the Distortion								
	*K* (MPa)	*ε*_0_	*n*		*q*	*k*	*k*_1_	*k*_2_	*k*_3_	*k*_4_	*k*_5_	*L*	*k_L_*
Values	500	0.01	0.25		2	20	100	50	0.5	0.9	20	2.0	300

**Table 2:** Coefficients of yield function and hardening law for DP780 and EDDQ (Data from [6]).

Material	Yld2000-2d Yield Function										Swift Hardening Law		
	*α*_1_	*α*_2_	*α*_3_	*α*_4_	*α*_5_	*α*_6_	*α*_7_	*α*_8_	*m*		*K* (MPa)	*ε*_0_	*n*
DP780	0.946	1.022	1.015	1.000	1.011	0.968	1.010	1.006	6		1295	0.0008	0.142
EDDQ	1.014	1.118	0.931	0.892	0.904	0.811	1.029	0.918	6		538	0.0075	0.267

**Table 3:** Coefficients of HAH-2d models for DP780 and EDDQ.

Material	*k*	*k*_1_	*k*_2_	*k*_3_	*k*_4_	*k*_5_	*k_L_*	*L*	*q*
DP780	45.0	135.2	39.6	0.475	1.0	0	0	1.0	2.0
EDDQ	12.80	773.9	295.9	0.551	1.0	0	205.9	2.226	2.0

**Table 4:** Prediction errors for DP780 under different two-step uniaxial tensions.

Conditions	RD 4%−TD	RD 4%–60°	RD 10%–60°	RD 4%–45°	RD 10%–45°
RMSE	20.11	24.07	37.85	24.49	47.13
AARE (%)	1.28	2.14	3.44	1.94	2.58

**Table 5:** Prediction errors for EDDQ under different two-step uniaxial tensions.

Conditions	RD 4%−TD	RD 4%–60°	RD 10%–60°	RD 4%–45°	RD 10%–45°
RMSE	5.59	8.65	9.86	4.74	5.40
AARE (%)	1.53	1.78	2.00	1.33	1.13

## Data Availability

Not applicable.

## Appendix A

Yld2000-2d anisotropic yield criterion [37] is defined as:

(A1) $$\phi ={\left|{X^{\prime }}_{1}-{X^{\prime }}_{2}\right|}^{m}+{\left|{X^{\prime \prime }}_{1}+2{X^{\prime \prime }}_{2}\right|}^{m}+{\left|2{X^{\prime \prime }}_{1}+{X^{\prime \prime }}_{2}\right|}^{m}=2{\bar{\sigma }}^{m}$$

where $\bar{\sigma }$ is the equivalent stress, ${X^{\prime }}_{i}$ and ${X^{\prime \prime }}_{i}$ (*i* = 1, 2) are the principal values of tensors $X^{\prime }=L^{\prime }\cdot \sigma$ and $X^{\prime \prime }=L^{\prime \prime }\cdot \sigma$, respectively. σ is Cauchy stress tensor. $L^{\prime }$ and $L^{\prime \prime }$ can be written in the following form

(A2) $$\left[L^{\prime }\right]=\left[\begin{array}{ccc} {L^{\prime }}_{11} & {L^{\prime }}_{12} & 0\\ {L^{\prime }}_{21} & {L^{\prime }}_{22} & 0\\ 0 & 0 & {L^{\prime }}_{66} \end{array}\right]=\frac{1}{3}\left[\begin{array}{ccc} 2\alpha_{1} & -\alpha_{1} & 0\\ -\alpha_{2} & 2\alpha_{2} & 0\\ 0 & 0 & \alpha_{7} \end{array}\right]$$

(A3) $$\left[L^{\prime \prime }\right]=\left[\begin{array}{ccc} {L^{\prime \prime }}_{11} & {L^{\prime \prime }}_{12} & 0\\ {L^{\prime \prime }}_{21} & {L^{\prime \prime }}_{22} & 0\\ 0 & 0 & {L^{\prime \prime }}_{66} \end{array}\right]=\frac{1}{9}\left[\begin{array}{ccc} 8\alpha_{5}-2\alpha_{3}-2\alpha_{6}+2\alpha_{4} & 4\alpha_{6}-4\alpha_{4}-4\alpha_{5}+\alpha_{3} & 0\\ 4\alpha_{3}-4\alpha_{5}-4\alpha_{4}+\alpha_{6} & 8\alpha_{4}-2\alpha_{6}-2\alpha_{3}+2\alpha_{5} & 0\\ 0 & 0 & 9\alpha_{8} \end{array}\right]$$

where $\alpha_{i}$ (*i* = 1, 2…8) are pending coefficients.

The principal values of X are

(A4) $$\left\{ \begin{array}{l} X_{1}=\frac{1}{2}\left(X_{11}+X_{22}+\sqrt{{\left(X_{11}-X_{22}\right)}^{2}+4X_{12}^{2}}\right)\\ X_{2}=\frac{1}{2}\left(X_{11}+X_{22}-\sqrt{{\left(X_{11}-X_{22}\right)}^{2}+4X_{12}^{2}}\right) \end{array}\right.$$

In the Yld2000-2d criterion, only the plane stress components, $\sigma_{xx}$, $\sigma_{yy}$ and $\sigma_{xy}$ are relevant, where *x* and *y* indicate the RD and TD, respectively. For the exponent *m* in Equation (A1), it is recommended to use 6 for BCC material and 8 for FCC material.
